# Robotic workflows for automated long-term adaptive laboratory evolution: improving ethanol utilization by *Corynebacterium glutamicum*

**DOI:** 10.1186/s12934-023-02180-5

**Published:** 2023-09-07

**Authors:** Lars Halle, Niels Hollmann, Niklas Tenhaef, Lea Mbengi, Christiane Glitz, Wolfgang Wiechert, Tino Polen, Meike Baumgart, Michael Bott, Stephan Noack

**Affiliations:** 1 Institute of Bio- and Geosciences, Forschungszentrum Jülich GmbH, IBG-1: Biotechnology, 52425 Jülich, Germany; 2 Bioeconomy Science Center (BioSC), Forschungszentrum Jülich GmbH, 52425 Jülich, Germany

**Keywords:** Adaptive laboratory evolution, Untargeted strain optimization, *Corynebacterium glutamicum*, Ethanol utilization, Acetaldehyde dehydrogenase, GlxR

## Abstract

**Background:**

Adaptive laboratory evolution (ALE) is known as a powerful tool for untargeted engineering of microbial strains and genomics research. It is particularly well suited for the adaptation of microorganisms to new environmental conditions, such as alternative substrate sources. Since the probability of generating beneficial mutations increases with the frequency of DNA replication, ALE experiments are ideally free of constraints on the required duration of cell proliferation.

**Results:**

Here, we present an extended robotic workflow for performing long-term evolution experiments based on fully automated repetitive batch cultures (rbALE) in a well-controlled microbioreactor environment. Using a microtiter plate recycling approach, the number of batches and thus cell generations is technically unlimited. By applying the validated workflow in three parallel rbALE runs, ethanol utilization by *Corynebacterium glutamicum* ATCC 13032 (WT) was significantly improved. The evolved mutant strain WT_EtOH-Evo showed a specific ethanol uptake rate of 8.45 *±* 0.12 mmol_EtOH_ g_CDW_^−1^ h^−1^ and a growth rate of 0.15 ± 0.01 h^−1^ in lab-scale bioreactors. Genome sequencing of this strain revealed a striking single nucleotide variation (SNV) upstream of the *ald* gene (NCgl2698, cg3096) encoding acetaldehyde dehydrogenase (ALDH). The mutated basepair was previously predicted to be part of the binding site for the global transcriptional regulator GlxR, and re-engineering demonstrated that the identified SNV is key for enhanced ethanol assimilation. Decreased binding of GlxR leads to increased synthesis of the rate-limiting enzyme ALDH, which was confirmed by proteomics measurements.

**Conclusions:**

The established rbALE technology is generally applicable to any microbial strain and selection pressure that fits the small-scale cultivation format. In addition, our specific results will enable improved production processes with *C. glutamicum* from ethanol, which is of particular interest for acetyl-CoA-derived products.

**Supplementary Information:**

The online version contains supplementary material available at 10.1186/s12934-023-02180-5.

## Background

Fossil raw materials such as crude oil, natural gas and coal are currently the most important energy sources on our planet. From 1970 to 2019, daily global oil consumption alone doubled from 7200 m^3^ to 15,500 m^3^ per day [1]. The largest sectors using petroleum are transport with a share of 54% and the chemical industry with 24%. However, fossil raw materials are finite and the non-circular value chain usually ends with the environmentally harmful greenhouse gas CO_2_. Therefore, new concepts and technologies to access alternative carbon sources and to close material cycles towards a modern and sustainable bio-based circular economy are intensively investigated [2–5]. A straightforward approach to close material cycles is the fixation of the released CO_2_. Taking into account external factors such as land usage, the electrocatalytic fixation of CO_2_ for the synthesis of low-molecular organic compounds such as ethanol could be a bio-economically viable option [6, 7]. For a sustainable material cycle in production processes, ethanol has the advantage that it is less complex to handle than C_1_ compounds such as methanol [8] and is easier to integrate as a carbon source in fermentation processes [9].

For example, the industrially used production organism *Corynebacterium glutamicum* [10, 11] possesses a natural pathway for ethanol metabolism [12]. Here ethanol is first oxidized to acetaldehyde via NAD-dependent alcohol dehydrogenase, followed by a second oxidation step to acetate via NAD-dependent acetaldehyde dehydrogenase. Finally, acetate is phosphorylated by acetate kinase and then CoA-activated by phosphotransacetylase to result in acetyl-CoA as central metabolic intermediate. Nevertheless, the usage of ethanol as feedstock for *C. glutamicum* is currently limited by its comparatively low tolerance, resulting in severe growth impairment at concentrations above 171 mM [12].

Direct targets for improving metabolic performance in biotechnological processes cannot always be predicted in the context of rational strain design. Adaptive laboratory evolution (ALE), which is based on the principle of evolutionary adaptation of a cell to changing environmental conditions, serves as an alternative approach here. This unique and powerful technology is increasingly used to exploit the high adaptability of rapidly dividing microorganisms for untargeted strain development and – in combination with genome sequencing – for genome research [13–16]. The combination of high-throughput ALE experiments with next generation sequencing, omics technologies, and genotype-to-phenotype studies leads to a deeper understanding of molecular mechanisms underlying complex phenotypes. Several successful examples of such information-rich ALE experiments have recently been demonstrated for biotechnologically relevant production organisms such as *Escherichia coli* [17–19], *Saccharomyces sp.* [20, 21], *Cupriavidus necator* [22], and *C. glutamicum* [23–25].

A recently introduced miniaturized and automated ALE approach is based on the Mini Pilot Plant (MPP) technology, which integrates the microbioreactor system BioLector® into a liquid handling platform [26, 27]. This setup allows for controlled repetitive batch cultivation in milliliter-scale and online monitoring of backscatter (BS)-based biomass, pH and dissolved oxygen. Sophisticated workflows employing the liquid handling robotics enable to perform all required process steps of the repetitive batch ALE (rbALE) in a fully automated manner. However, the established approach is still limited by the number of wells in the applied microtiter plate (MTP, e.g., 48-well FlowerPlate®) for the microbioreactor cultivations, restricting the duration of rbALE experiments. Since the probability of success of an ALE strongly depends on the number of generations, high throughput and unlimited duration methods are desirable.

In this study, we present the extension of the rbALE approach to perform long-term evolution experiments and demonstrate its successful application to improve ethanol utilization by *C. glutamicum*.

## Results and discussion

### Miniaturized and automated long-term rbALE

**Fig. 1:**
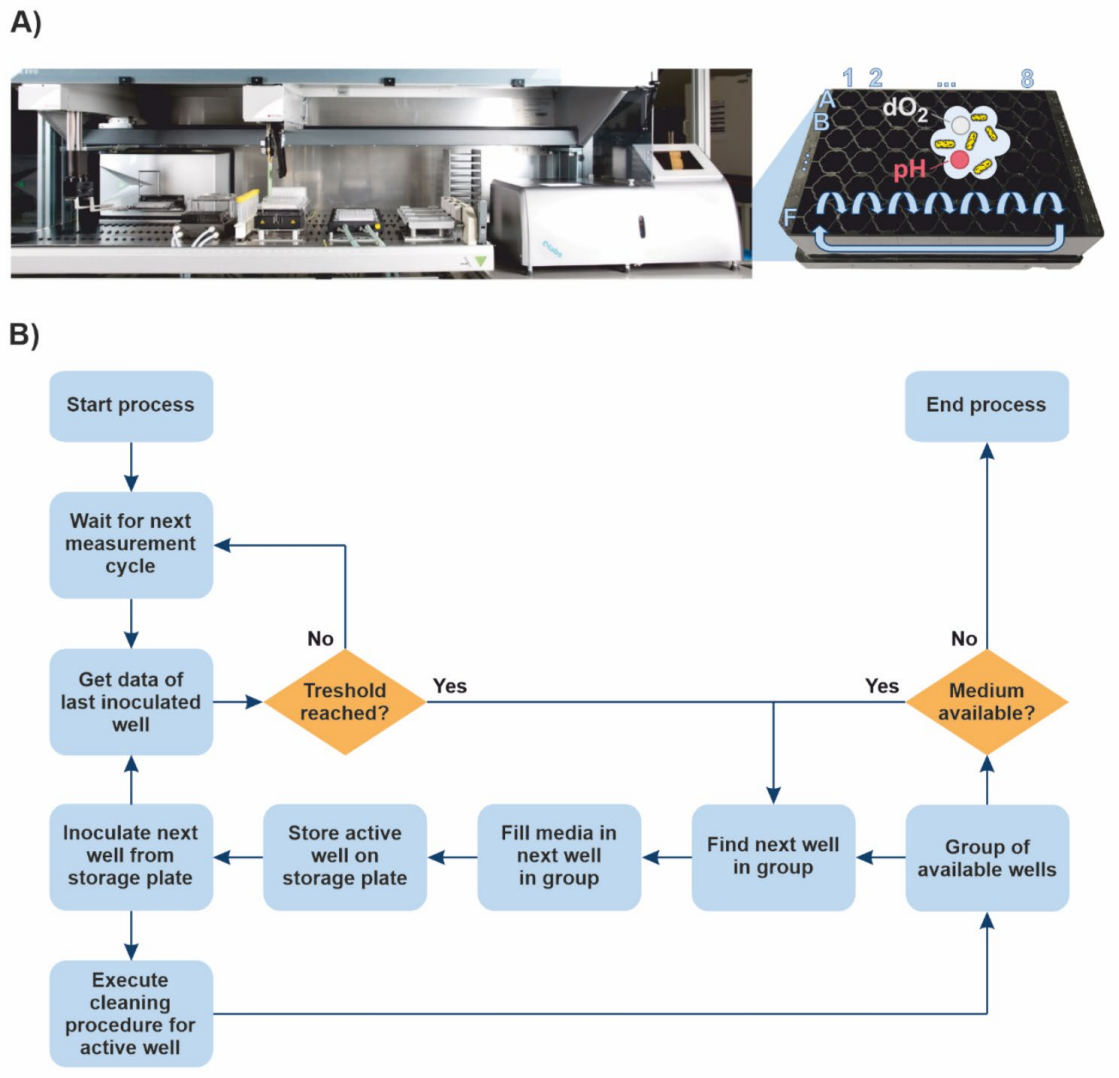
**Overview of the robotic workflow for automated long-term rbALE experiments. (A)** MPP technology which integrates the microbioreactor BioLector into a liquid handling system. On the right, a typical microtiter plate for cultivation in the micro-bioreactor is shown. The plate is divided into six groups (rows) of eight wells each. The repetitive batches scheme is shown with blue arrows. **(B)** Flow chart of the long-term rbALE process with well reuse. The process starts with the backscatter (BS) measurement cycle. The measurement data is evaluated after each cycle and when the BS threshold is reached, the inoculation of the next batch and the execution of the washing procedure is triggered

Current rbALE experiments in the MPP are limited by the number of wells in the used microtiter plate. To increase the number of generations and thus the probability of success for an rbALE experiment, the recently established repetitive batch approach by Radek et al. was further developed [26]. The original setup was based on performing repetitive batches along the six rows of a 48-well microtiter plate (Fig. 1A). For example, if six rbALE runs are performed in parallel, a maximum of eight consecutive batches is possible. Therefore, the simple idea to overcome this limitation was to reuse the wells within one evolution experiment. This reuse was enabled by emptying and cleaning the wells after each batch to create a condition that made them available for a new cultivation and allowed reliable BS measurement.

The developed workflow and setup for the long-term rbALE was as follows (Fig. 1B). The 48-well plate (FlowerPlate B, without optodes) was divided into six rbALE groups (rows A – F) with eight wells each. The six groups allowed a maximum of six parallel rbALE runs in one experiment. The applied cultivation medium was placed in 96-well deepwell plates (sealed with plastic foil with incisions) before the experiment and cooled to 4 °C on the platform cooling carrier. It should be noted here, that the cooling of the medium on the plate has a significant effect on the medium stability over time compared to storage at room temperature [26]. For cell preparation in the run-up, a pre-culture was performed in CGXII medium with 111 mM glucose in a shake flask. Cells were washed twice with sterile NaCl solution and diluted accordingly. The first well of each of the six rbALE groups was filled with 800 *µ*L medium and inoculated with 50 *µ*L of the washed cells to a starting optical density (OD_600_) of 0.2. The BS signal was measured every 15 min. Once the BS threshold (60% of the final BS, as determined from prior studies) was reached, the following process steps were executed (Fig. 1B):

1. 800 *µ*L of medium were transferred from one well of the 96-deep-well plate (media storage) to the next well in the group of the triggering well (48-well cultivation plate) and tempered for one measurement cycle (15 min) to allow the medium temperature to increase to the cultivation condition of 30 °C.
2. After temperature adjustment, the triggering well was completely harvested and stored on a sterile storage plate to ensure that the pipetting step for inoculation of the next well had the lowest possible error.
3. This inoculation step was then performed by pipetting 50 *µ*L of the harvested well from the storage plate to the 800 *µ*L of pre-tempered medium.
4. The harvested well was subsequently washed in two wash steps. For each step, 900 *µ*L of water was added and pipetted out again. After the second washing step, an additional pipetting step was performed for emptying to ensure that as much liquid as possible was removed from the well.

A residual volume of about 50 *µ*L, caused by the aspirating height of the robot of 1 mm above the plate bottom, could not be avoided. However, this residual liquid evaporated within the time that elapsed until the next inoculation of this well (min. 40 h). It should be noted that even if some liquid had been left behind during well recycling, in theory, this would not have affected the experiment. In the CGXII medium, all components are present in excess and the slight dilution effect would therefore not have had a significant limiting influence. Moreover, if cells were to survive in the residual fluid, these would not represent a contamination of an rbALE, but would simply be overgrown by the vital cells (exponential phase) of the next batch. In the best case, the even more extreme conditions in the residual liquid represent further selection pressure for higher stress tolerance. After the washing and drying process, the respective well was available again for another batch. As soon as the last well of the group (i.e. column 8 in Fig. 1A) was reached, the next batch was again performed in the first well of the group.

With the constant availability of a well for another batch, there is no longer a limit to the execution of the evolution experiment, and thus to the maximum number of generations possible. Furthermore, the number of generations per batch is independent of the growth rate and the organism used. It depends only on the inoculation density and the set threshold (cell density at this point). With the chosen setup, there are about three generations between the start and the end of each batch (start BS ≈ 8, threshold BS = 60). With this setup and without the developed extension, a maximum of 144 generations per experiment would be possible in a 48-well plate. With the extension, a total of 288 generations have already been generated in an experiment interrupted only by the experimenter. To further increase the number of generations per batch, one could consider reducing the inoculation density or increasing the substrate concentration (and the threshold adjusted to it). However, the latter carries the risk of undesirable oxygen limitation in an aerobic process. With lower cell density, the absolute number of replications per generation is lower, so the probability of beneficial mutations decreases.

Another strength of the extended setup is the higher degree of parallelization, for example to apply different selection strategies. The depicted experimental design now allows six replicates and no limit on the number of consecutive batches. A further increase in parallelization would also be conceivable by further shortening the rbALE groups.

### Validation of long-term rbALE workflow

**Fig. 2:**
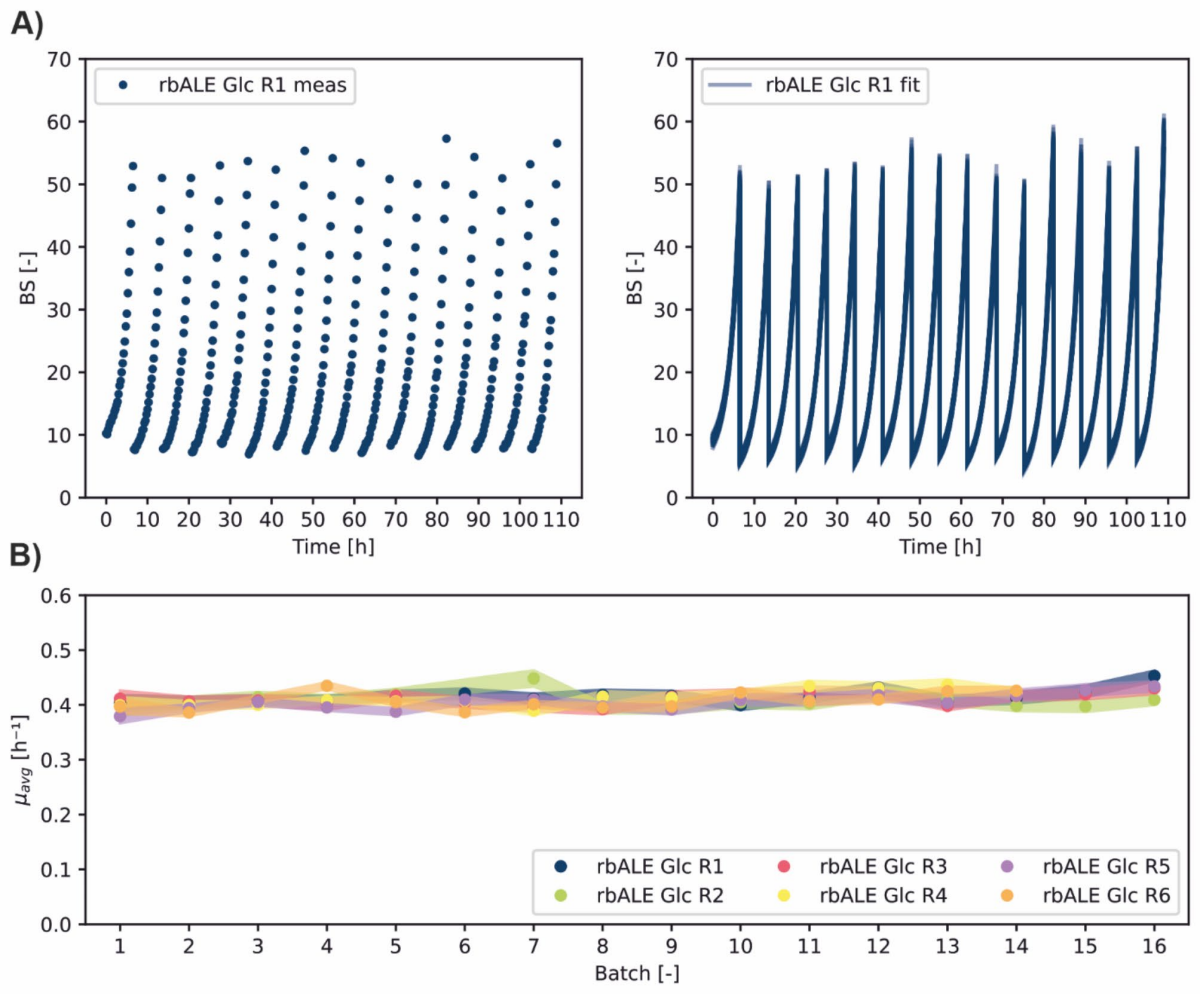
**Validation of long-term rbALE workflow. (A)** Example dataset rbALE Glc R1 out of six independent evolution experiments using the MPP technology. *C. glutamicum* WT was cultivated for a total number of 16 repetitive batches in one FlowerPlate and defined CGXII medium with 111 mM glucose as sole carbon and energy source. The resulting online BS measurements (meas) were used for process model fitting (fit). **(B)** Estimated specific growth rates across the single batches of all six rbALE runs

As a proof of concept for the developed long-term rbALE workflow, it was tested whether the reuse of previously used wells has an impact on growth performance under cultivation conditions without a selection pressure. Therefore, an rbALE experiment with *C. glutamicum* ATCC 13032 (wild-type strain, in the following abbreviated as WT) in CGXII medium with 111 mM glucose as sole carbon an energy source was performed. The obtained online BS measurements, simulated biomass trajectories and growth rate estimates for replicate R1 are depicted in Fig. 2A. In total, 96 single batches in only 5 days of cultivation showed stable growth performance across all batches. Moreover, in all eight independent rbALE runs the specific growth rate was almost constant across single batches (Fig. 2B). The average value of *µ* = 0.41 *±* 0.05 h^−1^ is in accordance with literature data for *C. glutamicum* WT [28].

### Improving ethanol utilization of *C. glutamicum* WT

In the following, a long-term rbALE experiment was performed to improve the ethanol utilization performance of *C. glutamicum* WT. As selection pressure, an ethanol concentration of 428 mM was defined. This condition was derived from a prior ethanol variation study in which *C. glutamicum* WT showed severely impaired growth at higher concentrations (data not shown). In addition to pure ethanol, mixtures of ethanol and glucose were also used. Two independent rbALE experiments in one FlowerPlate with three groups per condition were performed over a period of around 10 days and *>* 100 single batches per run. Of the resulting six rbALE runs, only the three runs with pure ethanol showed successful evolution of *C. glutamicum* WT, so the mixture experiments are not discussed further here.

**Fig. 3:**
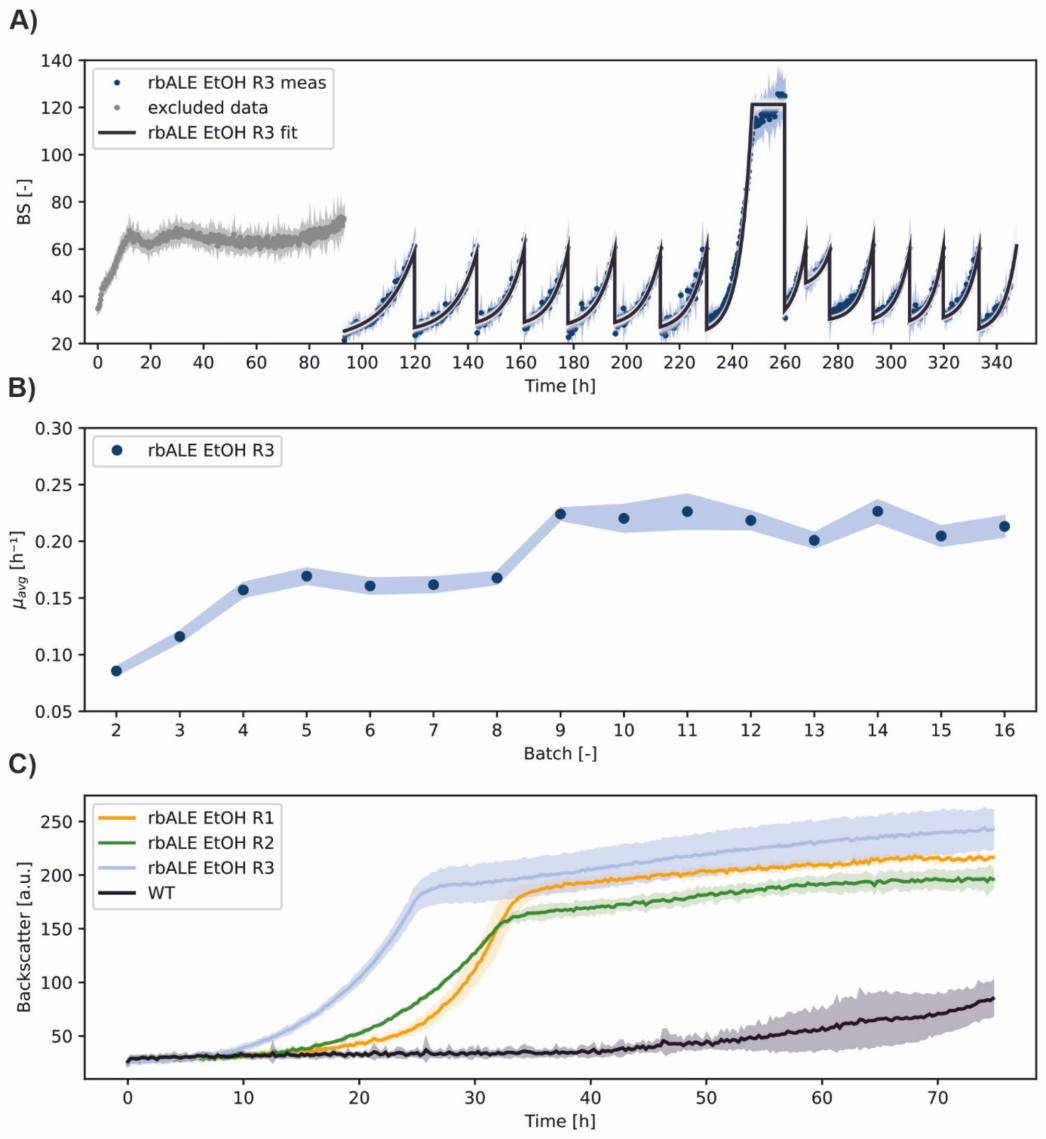
**Improving ethanol utilization of* C. glutamicum* WT through long-term rbALE. (A)** Example dataset rbALE EtOH R3 from a total of three replicate runs. *C. glutamicum* WT was cultivated for a total number of 16 repetitive batches in one FlowerPlate and defined CGXII medium with 428 mM ethanol as sole carbon and energy source. The resulting online BS measurements (meas) were used for process model fitting (fit). **(B)** Estimated specific growth rates across the single batches of rbALE EtOH R3. **(C)** Growth performances of the three evolved mutant cultures in comparison to *C. glutamicum* WT. Microscale batch experiments were performed under conditions equivalent to those of rbALE in three replicates per mutant strain. Mean values and standard deviations are shown as lines and shaded areas, respectively

As an example, the rbALE process EtOH R3 that led to an evolved *C. glutamicum* WT strain is shown in Fig. 3A (see Supplementary Fig. S1 for other replicates). The first batch showed an unexpected growth behaviour, which was probably due to an insufficient manual washing procedure between pre-culture on glucose and inoculation on ethanol. Therefore, this batch was excluded from the growth rate calculation. To avoid such an effect, the cell pellet should be washed with NaCl solution at least twice during a C-source change. The second batch of rbALE EtOH R3 exhibited an average growth rate of *µ* = 0.085 *±* 0.003 h^−1^ (Fig. 3B), which is in accordance with literature [12]. From the second batch onwards, a beneficial mutation event seemed to occur, leading to a steady, but wavy increase of the specific growth rate to *µ* = 0.16 *±* 0.01 h^−1^ until the fifth batch and a further increase to a final maximum specific growth rate of *µ* = 0.21 *±* 0.01 h^−1^. Unfortunately, the control system of the liquid handling robot stopped during the ninth batch due to a technical error and the culture continuously grew until the process was recovered. Inoculation of the subsequent batch from stationary phase cells also had no effect on further growth of the culture (cf. Figure 3A). As a workaround for such a failure, it is recommended to forward real-time information to the experimenter and permanently write a log file to restore the last experiment status.

The partially slow increase of the specific growth rate along the repetitive batches could be explained by the fact that only a small fraction of the cell population (only one cell at the beginning of the adaptive evolution) carries an advantageous mutation. For the mutant with *µ*_*evo*_*> µ*_*wt*_ to prevail, a certain number of generations must pass depending on the growth rate differences and these findings underline the necessity to perform long-term rbALE experiments. Similar trends were observed in the other replicates, i.e. random mutation events led to different dynamics of the BS signal, resembling growth competition between evolved mutants and non-evolved wild-type cells (cf. Supplementary Fig. S1).

To verify the successful adaptation of *C. glutamicum* WT to pure ethanol, the three evolved cultures and the wild type were subsequently characterized under equivalent cultivation conditions in one batch and three replicates each (Fig. 3C). All evolved cultures showed improved growth performance compared to the wild type. Nevertheless, there were remarkable differences in the lag phase and growth rates between the replicate runs of the rbALE experiments, pointing to different evolutionary states of the obtained mutants and thus their degree of adaptation to ethanol. The latter depends on the type, number, and timing of mutations or metabolic changes during rbALE, and further detailed analyses would be required to gain further insight here. The best performing mutant strain, denoted as *C. glutamicum* WT_EtOH-Evo, showed significantly improved growth performance on ethanol under microscale cultivation conditions.

### Verification of the growth performance of *C. glutamicum* WT_EtOH-Evo on ethanol

**Fig. 4:**
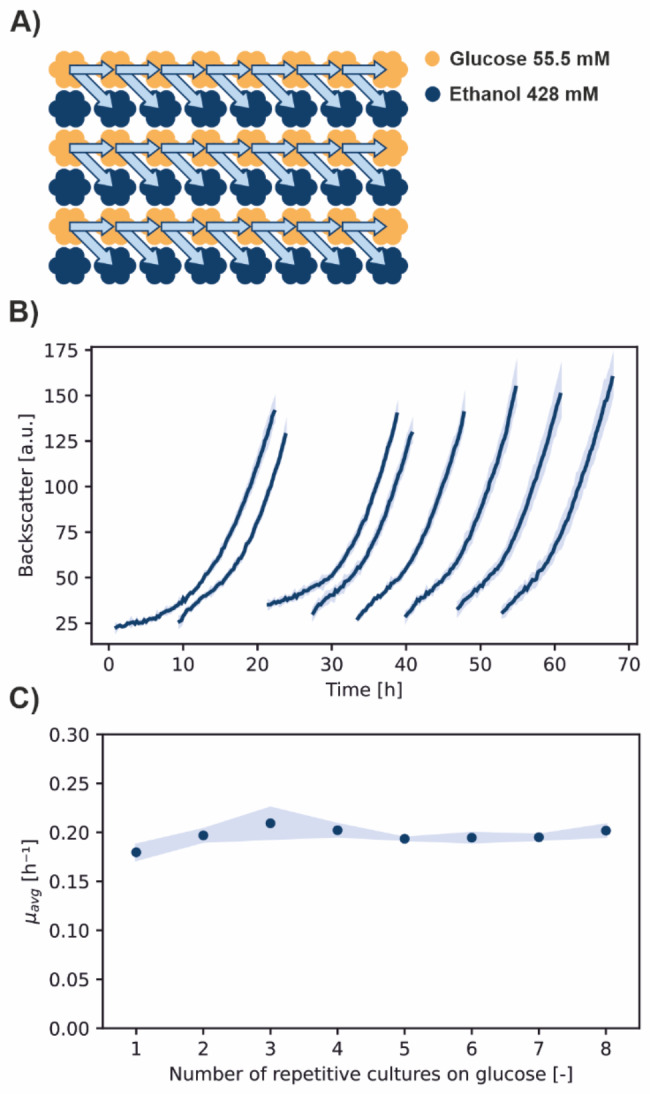
**Verification of evolved strain* C. glutamicum* WT_EtOH-Evo. (A)** Experimental design for automated microscale experiments using one FlowerPlate and defined CGXII medium with either 55 mM glucose or 428 mM ethanol as sole carbon and energy source, respectively. In triplicates, repetitive cultures were performed on glucose. After each glucose culture, a characterization on ethanol was performed. **(B)** Growth performances of *C. glutamicum* WT_EtOH-Evo in the 24 batch cultures on ethanol. Mean values and standard deviations were estimated from each of the three parallel cultures and are shown as lines and shaded areas, respectively. **(C)** Estimated specific growth rates across the batch cultures on ethanol

To verify the improved growth phenotype and genetic stability of the mutant strain on ethanol, a modified experimental setup of the described rbALE approach was used as described earlier [26]. Here, after a pre-culture in a shake flask on glucose (111 mM), the first column (A1 – F1) of a FlowerPlate was inoculated (Fig. 4A). Rows A, C, E contained CGXII medium with glucose (55.5 mM) and rows B, D, F contained CGXII medium with ethanol (428 mM) as the sole carbon and energy source. After reaching a BS threshold of 60% of the final BS, the next glucose well and the ethanol well below were inoculated from the glucose wells. In this way a series of repetitive cultures on glucose were performed before each ethanol batch. As a result, the specific growth rate of *C. glutamicum* WT_EtOH-Evo on ethanol was found to be repeatedly high (*µ* = 0.19 *±* 0.01 h^−1^), verifying the genetic stability of evolved ethanol utilization traits of this strain (Fig. 4C). It should be noted that the cells were not washed between carbon source changes. Therefore, a small amount of residual glucose is likely present at the beginning of growth on ethanol, but this does not affect the genetic stability result. As a corrective measure for possible inaccuracies, it is recommended to wash the cells in such a setup before inoculation.

### Key mutations identified by genomics and proteomics

**Fig. 5:**
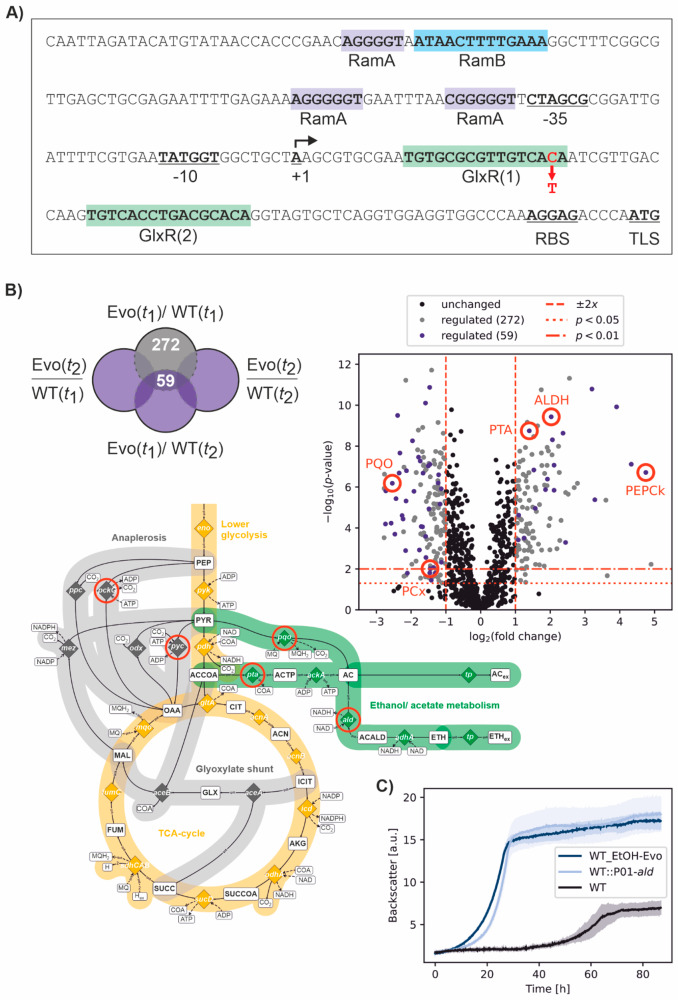
**Key mutation of *C. glutamicum *WT_EtOH-Evo identified by genomics and proteomics. (A)** Overview of *ald* (cg3096) promoter and regulatory elements. The transcriptional start site (TSS) is indicated with a black arrow and “+1” [30]. The − 10 and − 35 regions as well as the RBS and the translational start site (TLS) are underlined and written in bold letters. Regulator binding sites are shown as colored boxes. The mutation observed in strain WT_EtOH-Evo is indicated by a red arrow. **(B)** Differentially synthesized proteins of *C. glutamicum* WT_EtOH-Evo in comparison to the control strain *C. glutamicum* WT during cultivation in defined CGXII medium and 428 mM ethanol as sole carbon and energy source. Each culture was sampled twice, i.e. during exponential growth (EVO(*t*_1_), WT(*t*_1_)) and stationary phase (EVO(*t*_2_), WT(*t*_2_)), respectively. **Left**: Proteins with significant changes in all four sampled conditions were determined. **Right**: Volcano plot showing relative protein abundances between WT_EtOH-Evo and WT at first sampling point. Proteins that are significantly up or down regulated and can be associated with ethanol metabolism by *C. glutamicum* are highlighted in red. **(C)** Growth performance of the reengineered strain WT::P01-*ald* in comparison to WT_EtOH-Evo and the non-evolved wild type. Microscale experiments were performed using one FlowerPlate and defined CGXII medium with 428 mM ethanol as sole carbon and energy source. Mean values and standard deviations derived from six replicate cultures are shown as lines and shaded areas, respectively

To understand the effects of the genetic changes during evolution on ethanol, genomic DNA of WT_EtOH-Evo was isolated and sequenced using a MiSeq platform (Illumina). We discovered several single nucleotide variants (SNVs), which are listed in Supplementary Table S1. Most of the mutations had no obvious effect in the context of ethanol metabolism, because they were, e.g., silent mutations without any effect on the amino acid sequence or mutations within genes of unknown function or in genes with known function but without any connection to ethanol metabolism. Particularly striking was the SNV G to A 68 bp upstream of the monocistronic *ald* gene (NCgl2698, cg3096), encoding an NAD-dependent acetaldehyde dehydrogenase (ALDH). Transcription of *ald* starts 92 [29] or 93 bp upstream of the translational start site [30] (Fig. 5A). Expression of *ald* is regulated by at least three different transcriptional regulators, namely GlxR, RamA and RamB [29, 31]. GlxR (cg0350) is a homolog of the *Escherichia coli* cAMP receptor protein (Crp) and the global transcriptional regulator of carbon metabolism in corynebacteria [31]. Furthermore, GlxR is important for the regulation of other cellular processes, such as phosphate, sulfur, and iron homeostasis as well as stress responses. Two GlxR binding sites were predicted in the *ald* promoter, but only site GlxR(1) was bound by GlxR in electrophoretic mobility shift assays (EMSAs) [31] (Fig. 5A). Binding of the *ald* promoter by GlxR was also analysed by ChIP-chip and ChIP-Seq analyses in two independent studies [32, 33].

The two GlxR binding sites are located downstream of the transcriptional start site of *ald*, which suggests that *ald* is repressed by GlxR. The consensus motif for the GlxR binding site is TGTGA-N_6_-TCACA, while GTG and ACA seem to be of highest importance for high-affinity GlxR binding [32, 33]. The SNV G to A described above (please note that it is C→T in Fig. 5A because *ald* is encoded on the (-) strand) alters the GlxR(1) motif from TGTGCGCGTTGTCACA to TGTGCGCGTTGTCA**T**A, thereby presumably strongly inhibiting or even preventing GlxR binding. This should lead to an increased transcription of *ald* in WT_EtOH-Evo.

In fact, proteomic measurements directly comparing protein patterns of WT_EtOH-Evo and the WT grown on pure ethanol showed a 4-fold increased abundance of the ALDH protein in the evolved mutant strain (Fig. 5B). Other enzymes associated with increased ethanol catabolism in *C. glutamicum*, such as phosphotransacetylase (PTA) and phosphoenolpyruvate carboxykinase (PEPCk), were also strongly upregulated (Supplementary Table S2). In contrast, both pyruvate carboxylase (PCx) and pyruvate quinone:oxidoreductase (PQO) enzymes were significantly downregulated, possibly supporting the altered metabolism toward gluconeogenesis. The strong response of cells at the proteomic level upon switching from glucose to ethanol as a carbon and energy source was unexpected and will be studied in more detail in the future.

In *C. glutamicum* wild type, the specific activity of ALDH is half of that of alcohol dehydrogenase [12]. This suggests that the conversion of acetaldehyde to acetate could be a rate-limiting reaction step for ethanol assimilation (Fig. 5B, right). Most importantly, acetaldehyde is known as a highly reactive and growth-inhibiting intermediate. Therefore, an increased ALDH activity in WT_EtOH-Evo likely results in an accelerated conversion of acetaldehyde to acetate, thereby reducing the accumulation of the aldehyde at toxic levels and enhancing the carbon flux to acetate.

To confirm the impact of the single SNV upstream of the *ald* gene alongside the other SNVs detected, this mutation was reengineered in the wild type. The resulting strain WT::P01-*ald* showed a greatly increased growth performance in comparison to the WT (Fig. 5C), which is comparable to WT_EtOH-Evo, confirming that this particular mutation was the one responsible for the increased growth rate on ethanol of the evolved strain.

### Lab-scale cultivation of *C. glutamicum* WT_EtOH-Evo on ethanol

**Fig. 6:**
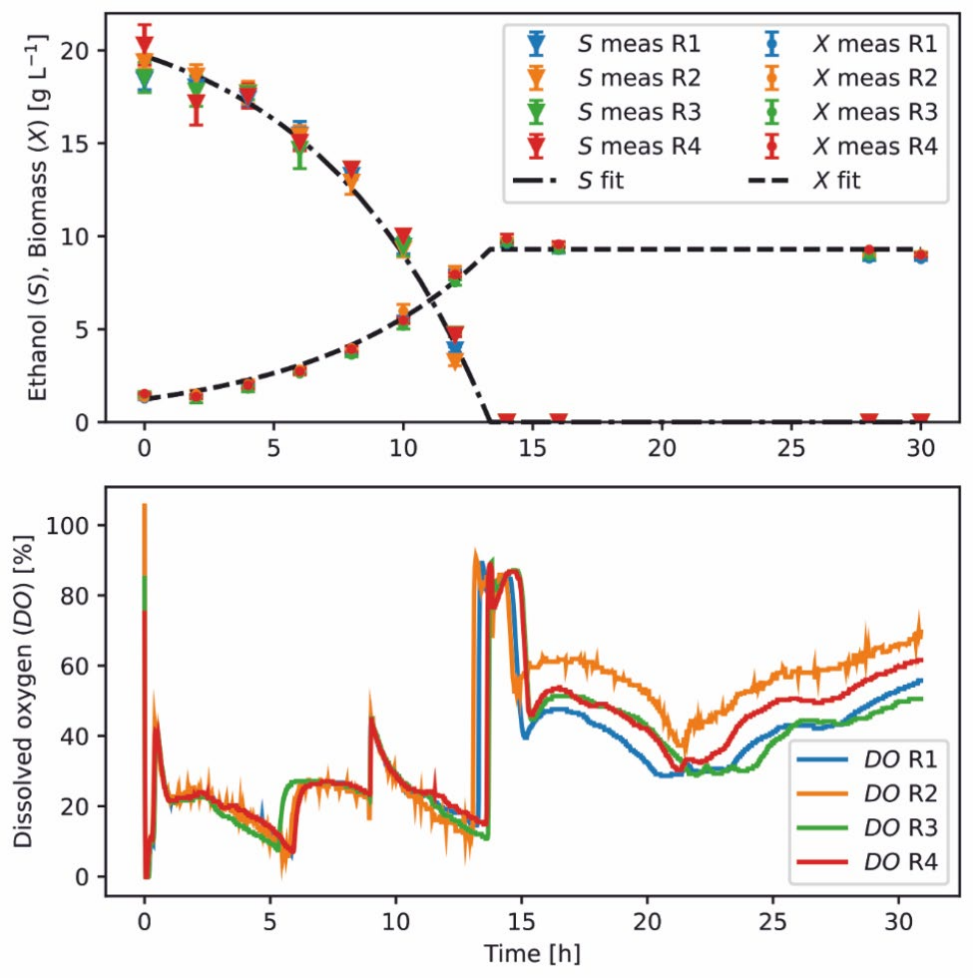
Cultivation of *C. glutamicum* WT_EtOH-Evo in lab-scale bioreactors. Batch experiments were performed in four parallel bioreactors using defined CGXII medium with 428 mM ethanol as sole carbon and energy source. Transient sampling was performed for offline ethanol and biomass measurements (meas) and the resulting data were used for process model fitting (fit)

A scale-up experiment was performed with WT_EtOH-Evo in 1-L bioreactors using defined CGXII medium and 428 mM ethanol as sole carbon and energy source (Fig. 6). Ethanol and biomass concentrations followed a classical batch profile. The first peaks in the DO signal after 6 and 9 h, respectively, are due to cascade-based control, where the maximum stirrer speed and gas volume flow rate were increased to avoid oxygen limitation. After 14 h of cultivation, ethanol was depleted, resulting in an abrupt change in cellular metabolism, as indicated by the starvation peak. Interestingly, after some time, a renewed respiration activity was observed without further biomass formation, and the cause of this effect is still unknown. Bioprocess modeling was applied to derive the following key performance indicators: *i*) maximum specific growth rate of *µ* = 0.15 *±* 0.01 h^−1^, which confirmed the growth performance of prior microscale experiments. However, the rate is lower than in the microbioreactor (*µ* = 0.19 *±* 0.01 h^−1^). This might be due to the CGXII medium used (without MOPS and urea) and the active gassing with air in the bioreactor; *ii*) specific biomass yield of *Y*_X/S_ = 0.45 ± 0.02 g_CDW_ g_EtOH_^−1^; and *iii*) specific ethanol consumption rate of *v*_upt_ = 8.45 *±* 0.12 mmol_EtOH_ g_CDW_^−1^ h^−1^.

## Conclusions

An extended workflow for automated, long-term rbALE experiments based on a MTP recycling procedure was presented. In terms of experimental time, the workflow can now compete with conventional lab-scale ALE applications based on chemostat cultures. The applicability and function of the long-term rbALE was demonstrated by successfully evolving wild-type *C. glutamicum* toward improved ethanol utilization. The resulting mutant strain *C. glutamicum* WT_EtOH-Evo showed non-delayed growth on ethanol as sole carbon and energy source with a specific growth rate of 0.15 *±* 0.01 h^−1^. Genome sequencing and proteomics revealed a mutation upstream of the *ald* gene that resulted in a 4-fold higher expression of the corresponding aldehyde dehydrogenase, presumably by interfering with binding of the transcriptional regulator GlxR, and hence a faster metabolization of ethanol into the central metabolism. Finally, the developed rbALE workflow provides a solid experimental basis for detailed studies on the dynamics of mutational events and enables more complex *in silico* process designs to further improve the control, speed and successful execution of ALE experiments.

## Methods

### Chemicals, bacterial strain and strain maintenance

All chemicals used in this work were purchased from Sigma Aldrich (Steinheim, Germany), Riedel-de Haën (Seelze, Germany) and Carl Roth GmbH (Karlsruhe, Germany). All strains and plasmids used in this study are listed in Table 1. All oligonucleotides used in this study are listed in Supplementary Table S3. For cultivation of *C. glutamicum*, defined CGXII medium [26] with either glucose or ethanol as carbon source was used. For strain maintenance, a working cell bank of *C. glutamicum* ATCC 13032 was established at the beginning of the work. For this purpose, 100 mL of cell suspension was grown in CGXII medium in a 1 L shake flask at 30 °C and 250 rpm in a thermo-shaker. Cells were washed twice with 0.9 g L^−1^ sterile NaCl solution and then suspended in 25% (w v^−1^) glycerol in 0.9 g L^−1^ sterile NaCl solution. The final OD_600_ was 89.7, and 20 cryotubes, each containing 1.6 mL, were prepared, and stored at *−* 80 °C. A cryotube was thawed and refrozen on ice a maximum of five times for preculture inoculation.

**Table 1:** Bacterial strains and plasmids used in this study

Strain or plasmid	Relevant characteristics	Source or Reference
*E. coli*		
DH5α	F^−^ Φ80*dlac*∆(*lacZ*)M15 ∆(*lacZYA-argF*) U169 *endA1 recA1 hsdR17* (r_K_^−^, m_K_^+^) *deoR thi-1 phoA supE44* λ^−^*gyrA96 relA1*; strain used for cloning procedures	[34]
***C. glutamicum***		
ATCC 13032 (WT)	Biotin-auxotrophic wild type	[35]
WT_EtOH-Evo	Derivative of ATCC 13032 with (among others) G→A mutation 68 bp upstream of the *ald* (cg3096) start codon resulting from ALE	This work
WT::P01-*ald*	Derivative of ATCC 13032 with G→A mutation is introduced 68 bp upstream of the *ald* (cg3096) start codon	This work
**Plasmids**		
pK19*mobsacB*	Kan^R^; plasmid for allelic exchange in *C. glutamicum*; (pK18 *ori*V_*E.c*_., *sacB*, *lacZ*α)	[36]
pK19*mobsacB*-P_01_-*ald*	Kan^R^; derivative of pK19*mobsacB* for mutating the promoter in front of *ald* (cg3096). The G→A mutation is introduced 68 pb upstream of the ALD start codon	This work

### Automated repetitive batch procedures

Repetitive batch cultivations were performed in the microbioreactor system BioLector® (Beckmann Coulter Life Sciences, CA, United States). 48-well FlowerPlates® (Beckmann Coulter Life Sciences, CA, United States) of the category BOH-1 (with optodes) were used during technical workflow validation and category B (without optodes) for the rbALE experiments. The use of BOH-1 plates is not an option for long-term experiments since the optode material degrades over time, resulting in significant disturbances of BS measurements [37]. All plates were covered with fleece foil to minimize evaporation during cultivation. All BioLector® cultures were performed at a shaking frequency of 1400 rpm, temperature of 30 °C, humidity of 85%, BS-gain of 20, and oxygen-ratio of 20.95% (air).

For the extended rbALE workflow the MTPs were divided into individual groups, each representing an independent rbALE run. The first well of each group was inoculated from a pre-culture in a shake flask as described above and normalized to a starting OD_600_ of 0.2. The BS signal was measured every 15 min. After the defined BS threshold of 60% of the maximum achievable biomass titer was reached, the next well in the group was filled with 800 *µ*L of fresh medium and inoculated with 50 *µ*L of cell suspension from the triggering well. This process continued until the last well of the group was inoculated. For details regarding the plate recycling approach see the results section.

### Long-term rbALE of *C. glutamicum* WT on ethanol

Based on previous cultivation data for wild-type *C. glutamicum* on ethanol, a substrate concentration of 428 mM (2.5% v v^*−*1^) was chosen for the rbALE experiment. Cells were first grown in a shake flask pre-culture containing CGXII medium with 111 mM glucose. The preculture was washed twice with sterile NaCl solution (0.9% w v^*−*1^). 800 *µ*L fresh medium was then inoculated with 50 *µ*L of the washed cell suspension in the first well of each ALE group (as described above). At the end of each rbALE run, samples were taken and frozen as glycerol culture in cryo-vials for subsequent analyses.

To test the growth performance of evolved strains, the samples containing the six mutants were thawed and grown as precultures in 4 mL CGXII medium and 111 mM glucose using a 24-well square-well plate. The plate was incubated at 250 rpm and 30 °C in the BioLector. Cells were washed twice with 4 mL sterile NaCl solution and diluted to obtain an OD_600_ of 3.4. 800 *µ*L medium was inoculated with 50 *µ*L of this cell solution to reach an initial OD_600_ of 0.2. For main batches, a BOH-1 plate was used and strains were cultivated in CGXII medium and 428 mM ethanol as sole carbon and energy source. To ensure validity of the phenotyping, each strain was cultivated in triplicate and the positions of the batches were randomly distributed on the plate to avoid any plate effect.

The basic data reading, e.g., plate-layout-files, was performed directly via Python 3.9.1 with the help of the package pandas [38]. The data from microbioreactor experiments was subsequently sorted, structured and measured BS values per batch normalized against the specific well before inoculation. The reading, structuring and assignment of the BioLector® data was done via the bletl package [39]. Numpy, Matplotlib and Seaborn were used for calculating, plotting and graphical visualization of the data [40–42]. All the code for data evaluation was written and executed in Jupyter notebooks (version 6.3.0) [43].

### Strain stability test

For testing strain stability, a cryo culture of WT_EtOH-Evo was thawed and a 100 mL preculture was inoculated in a 1 L shake flask on CGXII containing 111 mM glucose. The preculture was cultivated at 30 °C and 250 rpm. Cells were washed twice with sterile NaCl solution. The wells of the first row of the MTP were inoculated with 50 *µ*L cell suspension each in 800 *µ*L medium to an OD_600_ of 0.2. In this plate, the inoculated first column contained alternating medium with glucose (55.5 mM) and ethanol (428 mM). After the glucose-containing well had reached a defined threshold (60% of the final BS), the next well was filled with 800 *µ*L glucose medium (CGXII, 55 mM glucose) and inoculated with 50 *µ*L cell-suspension from the previous well. At the same time, the well in the row below was filled with 800 *µ*L ethanol medium (CGXII, 428 mM ethanol) and inoculated with 50 *µ*L cell-suspension from the previous well. In this way, the final batch on ethanol had already passed through eight precultures on glucose.

### Genomics and proteomics

For DNA sequencing of the evolved strain *C. glutamicum* WT_EtOH-Evo, cells were separated on an agar plate and a single colony was grown in brain heart infusion broth (BHI) (Carl Roth GmbH, Germany) in a 50 mL shake flask with 10% filling volume at 30 °C and 250 rpm. This preculture was used to inoculate a CGXII culture and the cells were harvested at the end of the exponential growth phase. After centrifugation, washing with sterile NaCl solution (0.9% w v^*−* 1^) and again centrifugation, the cell pellet was processed according to the instructions of the NucleoSpin Gel and PCR Clean-up kit (Macherey-Nagel, Germany) to extract the genomic DNA. The DNA extract was used for in-house sequencing using a MiSeq (Illumina).

For proteomics, *C. glutamicum* WT_EtOH-Evo and wild-type strain were grown in CGXII medium containing 171 mM ethanol in shake flask (triplicate) at 30 °C and 250 rpm and sampled at two time-points where cells were expected in exponential (Evo(*t*_1_) = 28.25 h, WT(*t*_1_) = 52.5 h) as well as stationary growth phase (Evo(*t*_2_) = 52.5 h, WT(*t*_2_) = 74.0 h), respectively. Cells were centrifuged for 10 min at 12,500 *g*, washed with NaCl solution (0.9% w v^*−*1^) and centrifuged again with the previous conditions. Cell pellets were suspended in lysis buffer containing 50 mM potassium phosphate buffer (pH 8.0), 2 mM EDTA, 2 mM DTT and supplemented with complete protease inhibitor cocktail (1697 498, Roche Applied Science, Basel, Switzerland). Cell suspensions were disrupted in a Precellys System (Bertin Instruments, Montignyle-Bretonneux FRANCE) using 0.1–0.2 mm glass beads and two glass beads with 5 mm diameter 3x for 30 s at maximum frequency. The supernatants containing protein fractions were collected and frozen at -20 °C until analysis. Protein concentration in crude extracts was determined by Bradford assay (B6916, Sigma Aldrich, USA) with BSA as standard. The resulting crude extracts were then used for untargeted LC-MS/MS measurements according to previously described methods [44], using an Infinity 1260 HPLC (Agilent Technologies) coupled to a Q-ToF 6600 mass spectrometer (Sciex, Darmstadt, Germany). Data acquisition and peak integration was carried out using the software PeakView 2.1 (Sciex), while protein identification was done with the software ProteinPilot 5.1 (Sciex). Marker View software (Sciex) was used for *t*-test analysis.

Further processing of protein data and functional annotation were performed in Python, using, among other standard packages, the library Biopython [45]. Differentially expressed proteins of *C. glutamicum* WT_EtOH-Evo in comparison to the control strain *C. glutamicum* WT were calculated for all four time-points, resulting in 272 (Evo(*t*_1_)/ WT(*t*_1_)), 118 (Evo(*t*_1_)/ WT(*t*_2_)), 343 (Evo(*t*_2_)/ WT(*t*_1_)), and 200 (Evo(*t*_2_)/ WT(*t*_2_)) significantly changed proteins, respectively (cf. Figure 5B). To distinguish between the specific proteomic response as a consequence of evolution and possible growth-dependent effects, an intersection of the four conditions studied was calculated, resulting in a reduced number of 59 regulated proteins (cf. Supplementary Table S2).

### Construction of plasmid pK19*mobsacB*-P_01_-*ald* and the strain WT::P_01_-*ald*

Plasmid pK19*mobsacB* was digested with fast digest restriction enzymes EcoRI and HindIII (Thermo Scientific, Schwerte, Germany) and purified using the DNA clean & concentrator kit (Zymo research, Irvine, CA, USA). The insert was amplified using Q5® High-Fidelity DNA Polymerase (NEB, Ipswich, MA, USA), oligonucleotides PLZM056/PLZM057, and a colony of strain WT_EtOH-Evo as template. The insert was purified using the Zymo DNA clean & concentrator kit. Both fragments were fused by Gibson assembly [46]. The reaction mix was used to transform chemically competent *Escherichia coli* DH5α [34] and the cells were plated onto lysogeny broth (LB) agar plates with 50 µg mL^−1^ kanamycin. Positive clones were identified by colony PCR with DreamTaq green PCR master mix (Thermo Scientific) and oligonucleotides M13-fw/M13-rv. Plasmids were isolated using Monarch® Plasmid Miniprep Kit (NEB), and sequenced by Eurofins genomics (Ebersberg, Germany) using the oligonucleotides M13-fw/M13-rv. pK19*mobsacB*-P_01_-*ald* was subsequently used to introduce the P_01_ mutation into the *C. glutamicum* wild type ATCC 13032 by double homologous recombination [36]. Chromosomal integration of the P01 mutation was confirmed by colony PCR with DreamTaq green PCR master mix and oligonucleotides P01-ald-Test-fw/P01-ald-Test-rv, and the mutant as a template. The PCR reaction was cleaned with the DNA clean & concentrator™ kit and sequenced by Eurofins genomics using the same oligonucleotides as for the PCR.

For growth phenotyping of the mutant strain WT_EtOH-Evo, the reengineered strain WT::P_01_-ald and the WT were picked as single cells from BHI agar plates and cultivated for seven hours in a 50 mL shake flask containing 5 mL BHI media (30 °C, 250 rpm). The 5 mL BHI media were transferred into a 500 mL shake flask containing 50 mL CGXII media with 111 mM glucose and incubated over night in a rotary shaker at 250 rpm and 30 °C. Cultures where centrifuged for 10 min at 4000 g and 4 °C, resuspended in sterile NaCl solution (0.9% w v^*−*1^) and centrifuged again with the previous conditions. The cell pellets where resuspended and diluted with sterile NaCl solution (0.9% w v^*−*1^) to inoculate 10 mL of CGXII media containing 428 mM ethanol with 100 µL inoculum to a starting OD of 0.2. From each 10 mL approach, six randomized wells on a FlowerPlate where filled with 900 µL for cultivation in a BioLector in six replicates to avoid any plate effects.

### Lab-scale bioreactor cultivations

For lab-scale-bioreactor cultivations with the evolved strain *C. glutamicum* WT_EtOH-Evo, four 1 L bioreactors (Eppendorf AG, Jülich, Germany) were assembled, pH electrodes calibrated, filled with 900 mL medium and autoclaved. Trace elements, biotin, protocatechuic acid (in total 25 mL stock solution) and ethanol (25 mL, 99.8% v v^*−*1^) were added to the bioreactor via syringe and port after autoclaving. The final ethanol concentration in the bioreactor was 428 mM, analogous to the microbioreactor experiment. As preculture, WT_EtOH-Evo was grown in six 1 L shake flasks at 30 °C and 250 rpm, each containing 200 mL CGXII medium and 111 mM glucose. Cells were washed twice with sterile NaCl solution (0.9% w v^*−*1^) and pooled. With each 50 mL of this preculture, all four bioreactors were inoculated to a starting OD_600_ of 3.5.

During the process, the pH was regulated at 7.0 via addition of phosphoric acid and ammonium hydroxide. A DO-dependent cascade was defined, in which the lower limit for the DO was set to 30%. The stirrer speed was first increased to a maximum of 1200 rpm and from 80% of this control value, the stirrer speed and gassing rate were further increased to 1400 rpm and 1 vvm, respectively, to meet the higher oxygen demand of the cells. For offline analytics, a sample of 7 mL was taken every two hours after mixing the sampling tube.

### Analytics for biomass and (by)-products

Cell dry weight (CDW) was determined in triplicates using 2 mL of the samples from bioreactor cultivations. After centrifugation for 10 min at 12,500 g in a tabletop centrifuge the supernatant was removed, and the cell pellet resuspended in 1 mL of sterile NaCl solution (0.9% w v^*−*1^). Following a second centrifugation step, the supernatant was discarded, and the cell mass was dried in a drying oven at 80 °C for 12–24 h. Samples were cooled down in a desiccator and weighed immediately after drying.

Optical density (OD_600_) was measured with a spectrophotometer (Shimadzu UV-1800, dual-beam device) at a wavelength of 600 nm in a 2 mL cuvette. The samples were diluted with phosphate-buffered saline (PBS) containing 137 mM NaCl, 2.7 mM potassium chloride and 12 mM total phosphate at pH 7.4. PBS also served as a reference for the measurement. The samples were diluted in highest 1 to 10 dilution steps until the measured OD_600_ was in the range between 0.05 and 0.3.

The remaining 5 mL bioreactor samples were centrifuged and the obtained supernatants sterile filtered with a syringe through a cellulose acetate filter (pore size 0.2 *µ*m, DIANielsen, Düren, Germany), and then diluted 1 to 2 with high purity water (MilliQ). The ethanol concentration was determined via HPLC on an Agilent 1100 Infinity system (Agilent Technologies, Santa Clara, CA). A polymer-based column for sugars and organic acids (Metab-AAC, 300 × 7.8 mm, BF-series, particle size 10 *µ*m, Isera, Düren, Germany) was used as the stationary phase and 100 mM H_2_SO_4_ as the mobile phase. The flow rate was 0.6 mL min^*−*1^ at 60 bar and the injection volume was 10 *µ*L. Ethanol was measured using a refractive index detector. As a reference, a dilution series of an ethanol standard starting from 1370 mM was prepared in seven 1 to 2 steps to 10.7 mM. The eight standard dilutions and all samples were measured in triplicate and averaged.

### Bioprocess modeling

Modeling of the rbALE experiments for growth rate estimation was performed using OpenModelica [47] in combination with the in-house python-based package Estim8 (unpublished). The object-oriented framework provides a series of classes and packages for building, implementing and applying bioprocess models in the form of ordinary differential equation systems. A simple rbALE model was formulated with biomass *X* and a substrate-independent specific growth rate *µ* = *µ*_*max*_ as model variables. The latter assumption was justified because experimental substrate concentrations clearly did not reach scale of affinity constants of wild-type *C. glutamicum* towards glucose and ethanol, respectively [48]. A linear calibration model of the form *X* = (*BS − b*)*/a* was applied to map the measured BS values to simulated cell dry weight. Transfer events were implemented to describe the switches between single batches of the rbALE runs by event triggered dilution of *X* based on a specific dilution factor *f*_*dil*_. Every *i*-th transfer event allowed switch to another growth rate *µ*_*i*_ to enable evolving growth rates during the simulation of the repetitive batches. While time-points for transfer events were automatically generated from the dataset, the remaining model parameters (*µ*_*i*_, *f*_*dil*_, *i*, *X*_0_, *a*, *b*) were estimated from the datasets employing state-of-the art routines for optimization and parameter uncertainty analysis [49, 50].

The data from the four bioreactor experiments were modelled with the software package pyFOOMB [51] and by applying classical Monod-kinetics for describing biomass growth and substrate consumption. Model parameters such as the maximum specific growth rate *µ*_*max*_, the Monod constant *K*_*S*_, and the biomass-specific substrate yield *Y*_*X/S*_ were introduced as global parameters along the four replicates. Initial values for biomass *X*_0_ and substrate *S*_0_ concentration were defined as local parameters since each bioreactor was started separately.

### Electronic supplementary material

Below is the link to the electronic supplementary material.

Supplementary Material 1

## Data Availability

The DNA sequence reads of the evolved mutant strain *C. glutamicum* WT_EtOH-Evo are available via the NCBI BioProject ID PRJNA987971. All other data generated or analysed during this study are included in this published article and its supplementary information files. Strains and plasmids generated during this study are available from the corresponding author upon request.

## Abbreviations

ALDH: Acetaldehyde dehydrogenase
ALE: Adaptive laboratory evolution
BS: Backscatter
CDW: Cell dry weight
cAMP: Cyclic adenosine monophosphate
Crp: cAMP receptor protein
EMSA: Electrophoretic mobility shift assay
fit: Process model fitting
meas: BS measurements
MPP: Mini Pilot Plant
MTP: Microtiter plate
PBS: Phosphate-buffered saline
rbALE: Repetitive batch ALE
SNV: Single nucleotide variant
TLS: Translational start site
TSS: Transcriptional start site
