# Supplementary material for: Robotic workflows for automated long-term adaptive laboratory evolution: improving ethanol utilization by Corynebacterium glutamicum
Source: Microb Cell Fact. 2023 Sep 7;22:175. doi: 10.1186/s12934-023-02180-5 (PMC10483779; doi:10.1186/s12934-023-02180-5)
Supplement: Supplementary file 1 — Supplementary Material 1 [file 12934_2023_2180_MOESM1_ESM.docx]

**Supporting Information**

**Robotic workflows for automated long-term adaptive laboratory evolution: Improving ethanol utilization by *Corynebacterium glutamicum***

Lars Halle^1,2^, Niels Hollmann^1^, Niklas Tenhaef^1^, Lea Mbengi^1^, Christiane Glitz^1^, Wolfgang Wiechert^1,2^, Tino Polen^1,2^, Meike Baumgart^1^, Michael Bott^1,2^, Stephan Noack^1,2,#^


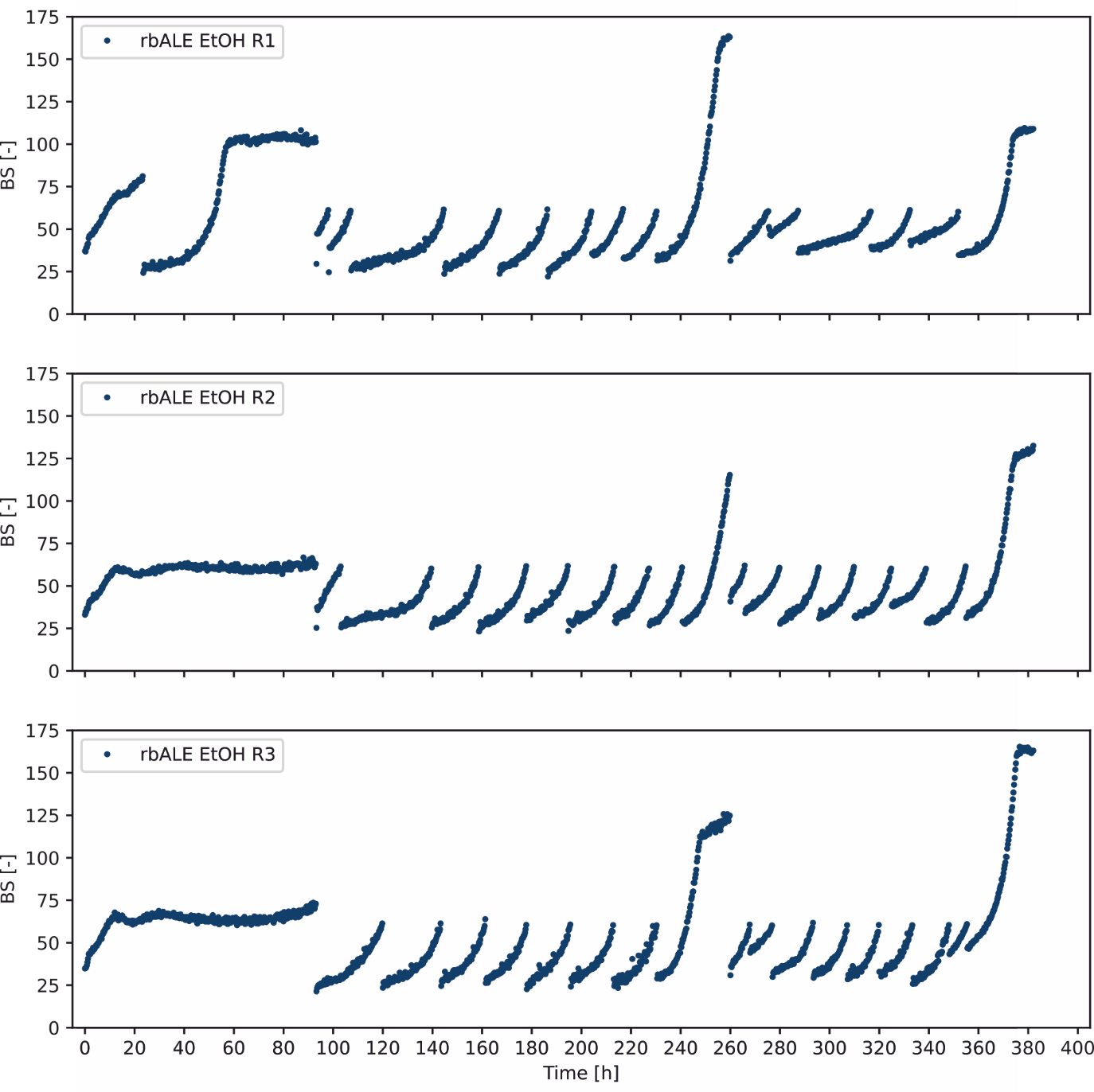


**Figure S1:** Long-term rbALE experiment to improve ethanol utilization of *C. glutamicum* WT. A total of three replicate runs in one FlowerPlate were performed. *C. glutamicum* WT was cultivated for a total number of 16 to 18 repetitive batches in one FlowerPlate and defined CGXII medium with 428 mM ethanol as sole carbon and energy source. The resulting online BS measurements against cultivation time are shown.

**
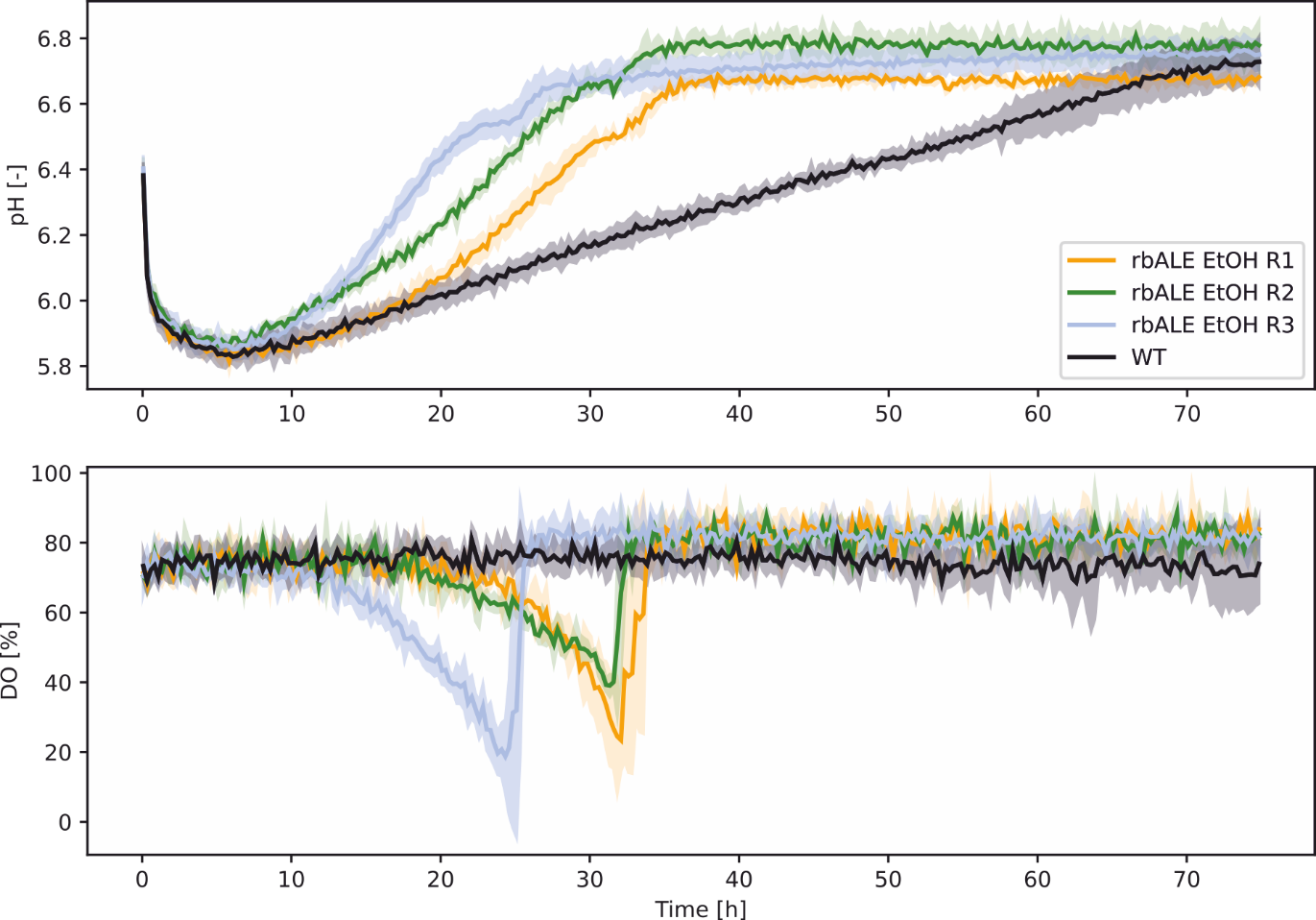
**

**Figure S2:** Additional online data (pH and dissolved oxygen (DO)) for the phenotyping experiments of the three evolved mutant strains in comparison to *C. glutamicum* WT (cf. Figure 3C).

**Table S1:** Single nucleotide variants (SNV) of rbALE EtOH R3 compared to the WT identified by genome sequencing appearing with a frequency of at least 75%.

| **Position and mutation on DNA level** | **Frequency (%)** | **Locus tag, gene name, annotation** | **Mutation and interpretation^a^** |
| --- | --- | --- | --- |
| 829516_SNV_T_A | 99.32 | cg0896, putative membrane protein | cg0896 Y967*, no promoter affected |
| 923955_SNV_G_T | 99.3 | cg0987, conserved hypothetical protein | cg0987 A12D, no promoter affected |
| 381944_SNV_T_G | 100 | P_cg0435_, *udgA1*, UDP-glucose 6-dehydrogenase | Mutation between RBS and TLS, 4 bp upstream of ATG, presumably no effect |
| 512738_SNV_G_A | 100 | P_cg0576_, *rpoB*, DNA-directed RNA polymerase β subunit | Mutation between TSS and TLS, 175 bp upstream of TLS, regulation not known. |
| 1096559_SNV_A_G | 100 | cg1189, hypothetical protein | cg1189 I38V, no promoter affected |
| 1813816_SNV_G_C | 100 | cg1935, *gntR2*, gluconate responsive repressor 2, involved in gluconate metabolism and the pentose phosphate pathway. | GntR2 V13L , no promoter affected |
| 1993795_SNV_T_C | 100 | NCgl1815, hypothetical protein, CGP4 | NCgl1815 R109R, silent mutation, no promoter affected |
| 1993797_SNV_G_T | 100 | NCgl1815, hypothetical protein, CGP4 | NCgl1815 R109R, silent mutation, no promoter affected |
| 1993809_SNV_A_G | 100 | NCgl1815, hypothetical protein, CGP4 | NCgl1815 L105L, silent mutation, no promoter affected |
| 1993912_SNV_C_T | 100 | NCgl1815, hypothetical protein, CGP4 | NCgl1815 P70P, silent mutation, no promoter affected |
| 2368804_SNV_A_C | 100 | cg2452, *galK*, putative galactokinase | cg2452 Q338P, no promoter affected |
| 2981767_SNV_G_A | 75.61 | P_cg3096_, *ald*, acetaldehyde dehydrogenase | Located in the GlxR binding site upstream of cg3096, presumably effect on transcription of *ald*. |

^a^RBS, Ribosome binding site; TLS, Translational start site; TSS, Transcriptional start site

**Table S2.** Differentially synthesized proteins of *C. glutamicum*WT_EtOH-Evo in comparison to the control strain *C. glutamicum*WT with ethanol as sole carbon and energy source. Proteins with statistically significant changes (*p*-value < 0.01, Log2(Fold-Change) > 1 for upregulated proteins and Log2(Fold-Change) < -1 for down-regulated proteins) as intersection of the four sampled conditions during exponential growth (EVO(*t*_1_), WT(*t*_1_)) and stationary phase (EVO(*t*_2_), WT(*t*_2_)) are shown (cf. Figure 5).

| **Identified protein** | **Gene** | **Locus** | **FC** | ***p*-value** |
| --- | --- | --- | --- | --- |
| Putative protein of nitroreductase-family |  | cg0404 | 0.15 | 6.39e-05 |
| Uncharacterized ACR, double-stranded β-helix domain |  | cg3277 | 0.151 | 1.55e-06 |
| Predicted nucleoside-diphosphate-sugar epimerase |  | cg3375 | 0.168 | 5.82e-05 |
| Pyruvate:quinone oxidoreductase | *pqo (poxB)* | cg2891 | 0.172 | 6.53e-07 |
| Conserved hypothetical protein |  | cg1131 | 0.181 | 0.0002 |
| Iron-regulated ABC transporter ATPase subunit | *sufC* | cg1762 | 0.185 | 3.69e-06 |
| O-Acetylhomoserine (Thiol)-Lyase | *metY* | cg0755 | 0.191 | 3.18e-10 |
| Uracil phosphoribosyltransferase | *upp* | cg0786 | 0.197 | 2.07e-07 |
| Electron transfer flavoprotein, α subunit | *fixB* | cg1387 | 0.2 | 2.18e-08 |
| Putative secreted protein |  | cg4005 | 0.209 | 2.47e-05 |
| Riboflavin synthase subunit β | *ribH* | cg1797 | 0.213 | 3.65e-05 |
| Fe-S cluster assembly protein | *sufB* | cg1764 | 0.224 | 0.00128 |
| Cu^2+^/cation-transporting ATPase transmembrane protein | *copB* | cg3281 | 0.226 | 4.81e-06 |
| 6-phosphogluconolactonase | *pgi (devB)* | cg1780 | 0.228 | 1.6e-07 |
| Citrate uptake transporter | *tctC* | cg3127 | 0.232 | 0.0161 |
| Putative membrane-bound protease modulator | *ppmA* | cg3138 | 0.257 | 5.51e-09 |
| Putative alkanal monooxygenase α chain, FMN-linked |  | cg2538 | 0.265 | 1.62e-06 |
| Electron transfer flavoprotein, β subunit | *fixA* | cg1386 | 0.29 | 3.46e-08 |
| Putative homoserine O-acetyltransferase |  | cg0961 | 0.295 | 0.00119 |
| Cysteinyl-tRNA synthetase | *cysS2* | cg1709 | 0.3 | 5.34e-08 |
| Peptide methionine sulfoxide reductase | *msrA* | cg3236 | 0.306 | 8.49e-05 |
| 30S ribosomal protein S10 | *rpsJ* | cg0593 | 0.314 | 8.78e-05 |
| High affinity ABC-type methionine transporter | *metQ* | cg0737 | 0.317 | 2.1e-07 |
| Bifunctional 3,4-dihydroxy-2-butanone 4-phosphate synthase/ GTP cyclohydrolase II protein | *ribA* | cg1798 | 0.333 | 7.86e-11 |
| 2-methycitrate dehydratase | *prpD2* | cg0759 | 0.334 | 0.000257 |
| Homoserine kinase | *thrB* | cg1338 | 0.336 | 0.00277 |
| Putative protein, CsbD-family, probably involved in stress response | *cybD* | cg0282 | 0.352 | 9.33e-08 |
| Putative coenzyme F420-dependent N5,N10-methylene tetrahydromethanopterin reductase or related flavin-depende |  | cg2329 | 0.357 | 1.16e-05 |
| Thioredoxin reductase | *trxB* | cg3422 | 0.362 | 1.35e-11 |
| Putative Fe-S cluster assembly protein |  | cg1759 | 0.363 | 0.0157 |
| Putative lysine decarboxylase-family protein |  | cg1261 | 0.364 | 7.8e-08 |
| Pyruvate carboxylase | *pyc* | cg0791 | 0.365 | 0.00976 |
| Putative NADPH quinone reductase or Zn-dependent oxidoreductase |  | cg3405 | 0.374 | 0.0357 |
| Fe-S cluster assembly membrane protein | *sufD* | cg1763 | 0.391 | 0.00857 |
| Cysteine desulfhydrase | *sufU* | cg1760 | 0.404 | 0.0161 |
| N-acetylglucosaminyl transferase | *murG* | cg2369 | 0.429 | 6.36e-07 |
| 30S ribosomal protein S19 | *rpsS* | cg0599 | 0.433 | 2.51e-07 |
| ATP-dependent helicase PCRA | *pcrA* | cg0976 | 0.444 | 6.82e-06 |
| 30S ribosomal protein S17 | *rpsQ* | cg0604 | 0.456 | 1.22e-06 |
| Oxaloacetate decarboxylase | *odx* | cg1458 | 0.457 | 0.0047 |
| Cytochrome aa3 oxidase, subunit 1 | *ctaD* | cg2780 | 2.116 | 0.000383 |
| Conserved hypothetical protein |  | cg0935 | 2.136 | 2.38e-05 |
| Hypothetical protein |  | cg0452 | 2.379 | 0.00031 |
| Phosphate acetyltransferase | *pta* | cg3048 | 2.637 | 1.79e-09 |
| Thiosulfate sulfurtransferase | *thtR* | cg0803 | 2.886 | 1.47e-07 |
| Cysteine desulfurase-like protein involved in Fe-S cluster assembly | *nadS* | cg1214 | 2.965 | 1.1e-05 |
| Putative metallo-β-lactamase superfamily protein |  | cg0349 | 3.641 | 1.34e-06 |
| Conserved hypothetical protein |  | cg2464 | 3.788 | 4.09e-07 |
| Aldehyde dehydrogenase | *ald* | cg3096 | 4.082 | 3.71e-10 |
| Putative signal-transduction protein containing cAMP-binding and CBS domain |  | cg1456 | 4.18 | 4.92e-09 |
| 4-hydroxy-3-methylbut-2-enyl diphosphate reductase | *IpsH (lytB)* | cg1164 | 4.316 | 8.57e-08 |
| Conserved hypothetical protein |  | cg2363 | 4.316 | 1.44e-06 |
| Putative secreted protein |  | cg2949 | 5.16 | 2.34e-09 |
| Trehalose uptake system, ABC-type, bacterial extracellular solute-binding protein | *tusE* | cg0834 | 5.429 | 0.00051 |
| Quinolinate synthetase | *nadA* | cg1216 | 9.182 | 1.63e-11 |
| δ-aminolevulinate dehydratase | *hemB* | cg0512 | 9.736 | 4.19e-06 |
| Conserved hypothetical protein |  | cg1045 | 14.999 | 1.21e-10 |
| Putative NADPH-dependent FMN reductase |  | cg3223 | 20.12 | 7.7e-08 |
| Phosphoenolpyruvate carboxykinase | *pck* | cg3169 | 26.869 | 1.94e-07 |

**Table S3:** Oligonucleotides used in this study.

| **Oligonucleotide** | **Sequence (5’ → 3’) and properties^a^** |
| --- | --- |
| **Construction of plasmid pK19*mobsacB*-P01-*ald* and PCR-analysis of the resulting mutants** | |
| PLZM056 | **CAGCTATGACCATGATTACGC**CATCCACACCGAAAAAATCCAACG |
| PLZM057 | **TTGTAAAACGACGGCCAGTGAATT**TCATTGGGTCTCCTTTGGGCCAC |
| M13-fw | CGCCAGGGTTTTCCCAGTCAC |
| M13-rv | AGCGGATAACAATTTCACACAGGA |
| P01-ald-Test-fw | CGGGCCAGAACCGCTAGACG |
| P01-ald-Test-rv | GCCACCAATGTAGTTCTCGTAGCGC |

^a^ Overlaps for Gibson assembly are written in bold letters.
